# Self-Supervised WiFi-Based Identity Recognition in Multi-User Smart Environments

**DOI:** 10.3390/s25103108

**Published:** 2025-05-14

**Authors:** Hamada Rizk, Ahmed Elmogy

**Affiliations:** 1 Computers & Control Engineering Department, Faculty of Engineering, Tanta University, Tanta 31527, Egypt; hamada_rizk@ist.osaka-u.ac.jp; 2 Graduate School of Information Science and Technology, Osaka University, Osaka 565-0871, Japan; 3 RIKEN Center for Computational Science, Kobe 650-0047, Japan; 4 Faculty of Computer Engineering & Sciences, Prince Sattam Ibn Abdelaziz University, Alkharj 16273, Saudi Arabia; 5 Faculty of Engineering, Tanta University, Tanta 31527, Egypt

**Keywords:** WiFi sensing, identity recognition, self-supervised learning, smart environments, CSI, human recognition, AI agents

## Abstract

The deployment of autonomous AI agents in smart environments has accelerated the need for accurate and privacy-preserving human identification. Traditional vision-based solutions, while effective in capturing spatial and contextual information, often face challenges related to high deployment costs, privacy concerns, and susceptibility to environmental variations. To address these limitations, we propose *IdentiFi*, a novel AI-driven human identification system that leverages WiFi-based wireless sensing and contrastive learning techniques. *IdentiFi* utilizes self-supervised and semi-supervised learning to extract robust, identity-specific representations from Channel State Information (CSI) data, effectively distinguishing between individuals even in dynamic, multi-occupant settings. The system’s temporal and contextual contrasting modules enhance its ability to model human motion and reduce multi-user interference, while class-aware contrastive learning minimizes the need for extensive labeled datasets. Extensive evaluations demonstrate that *IdentiFi* outperforms existing methods in terms of scalability, adaptability, and privacy preservation, making it highly suitable for AI agents in smart homes, healthcare facilities, security systems, and personalized services.

## 1. Introduction

The advancement of smart environments has led to the increasing deployment of autonomous AI agents capable of interacting with human users in an adaptive and context-aware manner [1,2,3]. These intelligent systems must process and respond to human presence, actions, and behaviors with a high degree of accuracy to facilitate personalized services, real-time decision-making, and enhanced automation. Whether implemented in smart homes, healthcare facilities, security systems, or personalized service applications, precise human identification remains a fundamental requirement for enabling seamless interactions and ensuring system reliability [2].

In smart home automation, AI agents must distinguish between different occupants to dynamically adjust environmental settings such as lighting, temperature, and entertainment preferences based on individual user profiles [1,2,4]. In healthcare environments, AI-driven monitoring systems must differentiate between patients, caregivers, and medical staff to provide personalized medical assistance, rehabilitation tracking, and emergency response mechanisms. Similarly, security applications necessitate robust human identification to support access control, anomaly detection, and threat assessment, enabling AI-driven systems to make autonomous real-time security decisions. Furthermore, in retail and service industries, AI-enhanced automation enables customer identification, preference prediction, and personalized service delivery, thereby improving user experience and engagement. Despite the critical need for accurate human identification in these domains, existing vision-based solutions—although capable of capturing detailed spatial and contextual information—face substantial limitations, including high deployment costs, privacy concerns, and susceptibility to environmental variations such as lighting conditions, occlusions, and cluttered spaces [5,6]. These challenges pose significant obstacles to the scalability and practicality of vision-based approaches, particularly in privacy-sensitive environments where continuous visual monitoring is undesirable.

To address these challenges, wireless sensing using Channel State Information (CSI) has emerged as a non-intrusive, device-free, and privacy-preserving alternative to vision-based methods [7,8,9]. CSI-based sensing leverages WiFi signal variations to detect human presence, movement patterns, and identity-specific signal characteristics, eliminating the need for wearable devices or intrusive visual monitoring. However, existing WiFi-based human identification methods exhibit limited robustness in multi-user scenarios, where overlapping signal reflections introduce interference, making it challenging to accurately distinguish between individuals [10,11,12]. Furthermore, many contemporary AI models rely heavily on supervised learning, requiring large amounts of labeled training data, which is impractical in real-world scenarios [13]. This is because annotating CSI data is labor-intensive, making it challenging to train supervised models effectively.

Given these constraints, there is a pressing need for an AI-driven framework that integrates wireless sensing with advanced self-supervised learning techniques to enable scalable, privacy-preserving, and adaptive human identification. AI agents operating in dynamic, multi-user environments must possess the capability to autonomously extract identity-specific representations from CSI data, adapt to previously unseen users, and generalize across different environmental conditions—all without relying on extensive labeled datasets. This necessitates the development of self-supervised and semi-supervised learning frameworks that enhance the robustness, scalability, and adaptability of AI-driven human identification systems.

In this paper, we introduce *IdentiFi*, a novel WiFi-based human identification system that leverages contrastive learning in a self-supervised and semi-supervised fashion. The system is designed to learn discriminative representations entirely from unlabeled CSI datasets, using contrastive learning mechanisms and augmentation techniques to effectively model both temporal dependencies and identity-specific features in CSI data. To achieve this, *IdentiFi* applies a set of customized augmentation strategies specifically designed for CSI time-series data. These augmentations generate multiple diverse yet correlated views of the same signal, enabling the model to extract robust identity representations under varying environmental conditions. The system incorporates two key contrastive learning modules to process these augmented CSI views effectively. The temporal contrasting module captures robust temporal dependencies in CSI signals by enforcing a cross-view prediction task. In this approach, past latent features extracted from one augmented view are used to predict future representations of another augmented view. This mechanism ensures that the learned representations remain stable over time and are resistant to small perturbations or environmental variations. By forcing the model to learn how identity-specific movement patterns evolve, the temporal contrasting module enhances the system’s ability to generalize across different individuals and environments. The contextual contrasting module strengthens the system’s ability to differentiate between individuals by enforcing identity-specific constraints. This module ensures that representations belonging to the same individual remain tightly clustered in the feature space while increasing the separation between representations from different individuals. By contrasting identity features across different CSI samples, this module effectively reduces the impact of overlapping signal reflections, ensuring reliable performance in multi-user environments. To further improve performance in real-world scenarios, *IdentiFi* extends its self-supervised learning approach to a semi-supervised setting by introducing class-aware contrastive learning. The system first fine-tunes the encoder using a small set of labeled CSI samples, enabling it to learn refined representations. Once fine-tuned, the model generates pseudo-labels for the remaining unlabeled data, allowing for class-aware contrastive learning. This process ensures that samples of the same identity are maximally similar while increasing the separability between samples belonging to different individuals. This semi-supervised approach enables *IdentiFi* to maintain high identification accuracy even when only a small portion of labeled data are available.

The proposed system effectively tackles the major challenges associated with CSI-based human identification. The temporal contrasting module ensures that variations in human motion over time are consistently modeled, allowing the system to capture stable movement patterns across different environments. The contextual contrasting module mitigates multi-user interference by learning identity-specific features that remain robust even when signals from multiple individuals overlap. The challenge of limited labeled data is addressed through class-aware contrastive learning, which utilizes pseudo-labeling and supervised contrastive learning to maximize the utility of the available labeled data. By integrating self-supervised learning techniques, *IdentiFi* eliminates the need for large-scale manual annotations while maintaining high identification accuracy. Furthermore, the system’s reliance on CSI data instead of visual inputs ensures that it operates in a privacy-preserving and non-intrusive manner, making it well suited for applications such as smart homes, healthcare monitoring, and security systems. Through the integration of self-supervised representation learning, contrastive modeling, and semi-supervised fine-tuning, *IdentiFi* establishes a new benchmark for robust, adaptive, and privacy-preserving human identification using wireless sensing.

The key contributions of this work are as follows. First, we propose a novel integration of TS-TCC and Transformer-based architectures for real-time human identification in multi-occupant environments. Second, we develop advanced augmentation techniques tailored to CSI data to improve robustness and generalization. Third, we demonstrate scalability, privacy preservation, and adaptability in cluttered, dynamic indoor environments through extensive evaluations. The proposed *IdentiFi* system bridges the gap between privacy-preserving sensing and accurate, real-time human identification, setting a new benchmark for smart environments.

The rest of this paper is organized follows. Some works related to human localization, activity recognition, and identification are introduced in Section 2. An overview of the proposed system is presented briefly in Section 3.1. Section 3 presents the details of the proposed system. The presented system is assessed and evaluated in Section 4. Finally, some conclusions are drawn and future work is proposed in Section 5.

## 2. Related Work

WiFi CSI-based human sensing is gaining traction as a compelling alternative to traditional sensing methods, thanks to its non-intrusive, environmentally resilient, and device-free nature. Within this field, research has largely focused on three key areas: human localization, human activity recognition, and human identification. While recognizing human activities is essential, having precise location information is equally critical for timely intervention in emergencies. Additionally, human identification plays a vital role in personalized services and security applications. Therefore, developing a system that seamlessly integrates all three functions remains a significant yet challenging goal. This section provides a concise overview of key research efforts in these domains and explores the challenges associated with handling multiple users.

### 2.1. WiFi-Based Human Localization

Human indoor localization is a well-established research problem with applications spanning healthcare, smart homes, robotics, gaming, augmented reality (AR), and virtual reality (VR). It focuses on estimating user positions to enable seamless human–computer interaction and support various intelligent services [14,15]. Beyond simply determining a user’s location, localization systems provide context-aware information, such as movement patterns, behavioral insights, and environmental interactions, which are crucial for applications like elderly care monitoring [5,16], contact tracing [17] and intelligent navigation [18,19].

Various technologies have been explored to achieve reliable indoor localization, including ultrasound-based systems [20], which use acoustic signals for precise ranging but are affected by environmental noise and obstructions. Infrared-based tracking [21] is commonly used in motion capture systems but requires line-of-sight and is sensitive to ambient light interference. Magnetic-field-based localization [22,23,24] leverages ambient magnetic variations, which work well in specific indoor environments but struggle with spatial inconsistencies caused by infrastructure changes. Despite the progress in these technologies, they are often constrained by hardware limitations, environmental dependencies, and scalability issues. These challenges have led to a growing interest in WiFi-based localization, which takes advantage of the ubiquity of WiFi infrastructure to provide non-intrusive and device-free localization solutions [25,26,27].

The development of WiFi-based localization has progressed through multiple approaches. Early efforts relied on Naïve Bayes classifiers [28], employing received signal strength (RSS) fingerprinting, which provided acceptable accuracy but was highly susceptible to environmental changes and signal fluctuations [29,30,31]. To improve performance, researchers introduced Sparse Auto-encoders (SAEs) [25], leveraging CSI (Channel State Information) images for localization. However, its fully connected architecture resulted in a model with a large number of parameters, increasing computational complexity and requiring extensive labeled training data [8,32,33]. Further advancements led to the adoption of deep learning-based approaches, including Long Short-Term Memory (LSTM) networks [34] and Convolutional Neural Networks (CNNs) [14], which significantly improved localization accuracy. LSTMs proved effective at capturing temporal variations in CSI, while CNNs extracted spatial features from CSI amplitude and phase information, enhancing robustness against signal noise and multipath effects. Among these approaches, CNN-1D [14] has shown exceptional performance in WiFi-based localization by efficiently processing sequential and spatial signal attributes, leading to higher accuracy and faster inference times compared to traditional methods.

While WiFi-based indoor localization has seen significant advancements, several challenges remain. One major issue is scalability and generalization, as models trained in one environment often struggle in new, unseen locations due to variations in WiFi signal propagation [12,35]. Additionally, handling multiple users introduces interference, signal overlap, and complex CSI variations, requiring more sophisticated multi-user localization algorithms [12]. Reducing deployment costs is another critical challenge, as many WiFi-based systems rely on extensive fingerprinting efforts, which are time-consuming and labor-intensive. Recent research explores self-adaptive learning models to minimize manual calibration [36,37,38]. Furthermore, integrating WiFi CSI with complementary sensing technologies such as inertial measurement units (IMUs), LiDAR, and ultra-wideband (UWB) could enhance accuracy and robustness, enabling hybrid localization solutions for real-world deployment [7].

WiFi-based indoor localization has evolved from traditional statistical models to advanced deep learning techniques, demonstrating great potential in real-world applications. The integration of CNNs, LSTMs, and hybrid sensing technologies continues to push the boundaries of accuracy and reliability, paving the way for next-generation ubiquitous localization systems [12,39,40]. As research progresses, overcoming the existing challenges and refining multi-modal localization techniques will be crucial for achieving seamless and highly accurate human identification in diverse indoor environments.

### 2.2. WiFi-Based Human Activity Recognition (HAR)

The field of WiFi-based Human Activity Recognition (HAR) has gained significant attention due to its ability to analyze user behaviors with greater detail than traditional identification and localization tasks. Unlike wearable-based or vision-based HAR systems, WiFi-based HAR is device-free, non-intrusive, and environmentally robust, making it an attractive solution for applications in smart homes, healthcare monitoring, security surveillance, and human–computer interaction [41]. By leveraging CSI from WiFi signals, these systems can detect fine-grained human activities, such as walking, sitting, hand gestures, and even respiration patterns, without requiring the user to carry any devices.

Early HAR approaches primarily relied on handcrafted feature extraction, where researchers manually designed statistical and frequency-domain features from CSI data to classify activities using machine learning models like Support Vector Machines (SVMs) and Random Forests [41]. However, these methods often struggled to generalize across different environments, individuals, and device settings due to the high variability of WiFi signals. The inherent limitations of manually extracting meaningful features led to a shift towards deep learning-based approaches, which automatically learn discriminative representations from raw CSI data. To overcome these limitations, researchers began adopting Long Short-Term Memory (LSTM) networks [41] and Convolutional Neural Networks (CNNs) [33,42,43], which have proven highly effective in extracting meaningful features from CSI. LSTMs, a type of recurrent neural network (RNN), excel in capturing temporal dependencies within CSI time-series data, making them particularly useful for HAR tasks that involve sequential movements, such as walking or running [41]. Meanwhile, CNNs are well suited for extracting spatial features from CSI amplitude and phase information, allowing them to identify fine-grained motion patterns that distinguish between different activities [33,42,43]. A major breakthrough in WiFi HAR came with the development of CNN-LSTM hybrid models [8,26,44], which effectively combine the strengths of CNNs for spatial feature extraction and LSTMs for temporal modeling. These hybrid architectures have demonstrated superior performance by capturing both short-term and long-term motion variations, enabling more robust activity recognition even in complex indoor environments [8,26,44,45].

Despite the advancements in CNN-LSTM architectures, WiFi-based HAR systems still face challenges in generalization across different environments and hardware configurations. Variations in WiFi signal reflections, device placements, and environmental obstacles can lead to inconsistent CSI data, reducing classification accuracy. To mitigate these issues, researchers have integrated Generative Adversarial Networks (GANs) [9] with CNNs to introduce adversarial learning, improving model robustness by generating synthetic CSI data that mimic real-world variations. This technique allows HAR models to become more resilient to domain shifts, ensuring consistent performance across different settings [9]. Another significant advancement is the incorporation of attention-based models, such as ABLSTM (Attention-Based BiLSTM) [7,46], which enhance HAR performance by allowing the model to focus on the most informative parts of the CSI sequence. By dynamically assigning weights to different time steps, attention mechanisms improve the recognition of subtle motion variations, leading to better classification accuracy for similar activities (e.g., sitting vs. standing) [7,46].

The latest innovation in WiFi-based HAR is the adoption of Transformer-based architectures, particularly the Convolution-Augmented Transformer (THAT) [43], which integrates multi-scale CNNs with attention layers. Transformers have revolutionized natural language processing (NLP) due to their ability to model long-range dependencies, and their application to HAR has yielded state-of-the-art results [43]. The THAT model capitalizes on CNNs’ ability to extract hierarchical spatial features while leveraging self-attention mechanisms to capture global temporal dependencies, significantly improving HAR accuracy. Compared to traditional CNN-LSTM models, Transformer-based HAR systems demonstrate higher robustness to noise, better adaptability across different environments, and improved efficiency in processing large-scale CSI data. This innovation marks a crucial step toward deploying real-world WiFi-based HAR systems in smart environments, security applications, and healthcare monitoring [43].

Despite these advancements, several challenges remain in WiFi-based HAR. One major issue is scalability and generalization, as models trained in one environment often fail to generalize to new settings due to differences in WiFi signal propagation. Additionally, handling multiple users introduces interference, signal overlap, and complex CSI variations, requiring more sophisticated multi-user localization algorithms [42,47]. Low-latency real-time processing is another critical challenge, as deploying HAR models in real-world applications demands efficient inference speeds to ensure real-time activity recognition [12,47]. Finally, privacy and security concerns remain an important consideration, as passive WiFi sensing raises ethical questions regarding unauthorized user tracking and data privacy.

### 2.3. WiFi Human Identification

User identification in indoor environments using WiFi has emerged as a promising alternative to traditional biometric authentication methods, such as fingerprint scanning, facial recognition, and gait analysis. Unlike conventional methods, which require direct user interaction with a sensing device, WiFi-based identification is device-free, non-intrusive, and privacy-preserving, making it highly suitable for applications in smart homes, healthcare facilities, security systems, and personalized services [43,48]. By leveraging CSI, which captures unique signal perturbations caused by a person’s movement and body structure, WiFi-based systems can differentiate individuals based on their distinctive signal patterns.

Early approaches to WiFi-based user identification primarily relied on handcrafted features, where statistical and frequency-domain features were extracted from CSI data and classified using traditional machine learning models like SVMs, k-NN, and Random Forests [41]. However, these methods struggled to generalize across different environments and were highly sensitive to variations in WiFi signal propagation. To overcome these limitations, researchers adopted deep learning-based approaches, such as CNNs and LSTM networks, which automatically learn spatial and temporal patterns from raw CSI data. CNNs excel at capturing spatial signal characteristics, while LSTMs are effective in modeling long-term dependencies in movement sequences, making them ideal for distinguishing individuals based on their walking patterns and body-induced signal distortions.

More recently, hybrid deep learning models and attention-based architectures have significantly improved user identification accuracy in indoor WiFi environments. For instance, CNN-LSTM hybrids leverage CNNs for spatial feature extraction and LSTMs for sequential modeling, enhancing the system’s ability to recognize user-specific motion dynamics [49,50]. Additionally, Transformer-based models, such as the Convolution-Augmented Transformer (THAT), have introduced self-attention mechanisms, allowing the model to focus on the most distinguishing features in CSI data while filtering out irrelevant noise [51]. Another major advancement is the integration of Generative Adversarial Networks (GANs), which generate synthetic CSI samples to improve model robustness and adaptability to different environmental settings [52]. These advancements bring WiFi-based user identification closer to real-world deployment, making it a scalable, efficient, and secure solution for indoor authentication and personalized service applications.

Despite the above-mentioned achievements and advancements in using WiFi for indoor human identification, there are still many challenges. Using WiFi for indoor human identification presents several challenges, primarily related to signal variability, interference, and environmental dependencies. WiFi signals are highly sensitive to obstacles, reflections, and multipath effects, leading to inconsistencies in RSS and CSI. These variations make it difficult to achieve stable and accurate identification across different environments. Additionally, scalability remains a challenge, as models trained in one location often struggle to generalize to new spaces due to changes in WiFi signal propagation. The presence of multiple users further complicates identification, as overlapping signals and movement patterns introduce noise and ambiguity. Moreover, deploying WiFi-based identification systems typically requires extensive fingerprinting and calibration efforts, which are time-consuming and labor-intensive. To overcome these challenges, researchers are exploring adaptive learning models, multi-modal sensor fusion, and more robust deep learning architectures to enhance identification accuracy and system reliability in dynamic indoor settings. A summary of these challenges and some suggested solutions can be seen in [11].

## 3. *IdentiFi* System Details

### 3.1. *IdentiFi* System Overview

**Figure 1:**
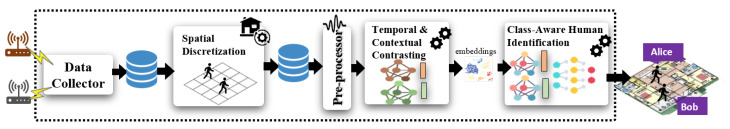
*IdentiFi* system architecture.

Figure 1 illustrates the architecture of *IdentiFi*, the proposed CSI-based Human Identification system, which is designed to determine whether a person is present in a specific spatial cell and, if so, to accurately identify them using fine-grained CSI data.

The process begins with the **data collection** module, which captures raw CSI signals as users perform natural activities in different locations within an indoor environment. This module integrates both hardware components—such as transmitters and receivers—and control software to ensure synchronized and reliable data collection. These raw signals reflect subtle changes in the wireless channel caused by human presence and movement.

Following data collection, the **spatial discretization** module divides the environment into a virtual grid, assigning each spatial region to a cell. During data recording, each CSI sample is tagged with the cell label corresponding to the user’s position. This enables the system to learn spatially grounded patterns and associate signal characteristics with physical locations. The **preprocessing** module prepares the CSI data for training by applying signal cleaning techniques such as noise reduction and normalization. It also structures the data into fixed-length sequences, facilitating consistent temporal analysis and efficient input to the model. At the heart of the system lies the **temporal and contextual contrasting (TCC)** module, which performs unsupervised representation learning. This module is responsible for extracting robust and generalizable features from unlabeled CSI sequences (inspired by this framework [53]). It begins by generating two semantically consistent but augmented views of the same input using a combination of strong and weak perturbations. These augmentations simulate various environmental conditions, encouraging the model to learn invariant features related to human presence and identity. The **temporal contrastive** submodule plays a key role in capturing time-dependent characteristics of human motion. It uses two autoregressive models trained in a cross-view prediction setup—each model attempts to predict the future of one view using the past of the other. This cross-temporal prediction task forces the system to model temporal dependencies that are crucial for distinguishing individuals based on their movement signatures. Complementary to this, the **contextual contrastive** submodule aligns the context representations produced by both views. By maximizing the agreement between corresponding context embeddings, it ensures temporal and semantic consistency across different views. Together, the temporal and contextual contrastive components form a dual-view self-supervised framework that serves as the foundation for downstream tasks such as human identification.

To enable *IdentiFi* to operate effectively under limited labeled data, we introduce the **class-aware human identification** module, which transitions the framework into a semi-supervised learning paradigm. This extension refines the previously learned features to be identity-aware through a four-phase training pipeline. In the first phase, the encoder is randomly initialized and trained using the self-supervised TCC module on unlabeled CSI data. This step helps the model learn general-purpose, context-rich representations without requiring any identity labels. In the second phase, the pretrained encoder is fine-tuned using a small set of labeled CSI samples, enabling the model to adapt its features to identity-specific information and improve its ability to discriminate between individuals. The third phase involves generating pseudo-labels for the remaining unlabeled data using the fine-tuned encoder. These pseudo-labels serve as an extended training set, allowing the system to leverage a much larger dataset without additional annotation efforts. Finally, in the fourth phase, the model is trained on both labeled and pseudo-labeled data using supervised contrastive loss. During this phase, the unsupervised contextual contrastive loss is replaced with a supervised version that explicitly encourages the model to group samples of the same identity together and push apart those of different identities. This class-aware semi-supervised strategy significantly enhances the model’s performance in real-world environments, where labeled data are scarce and environmental dynamics vary. By combining strong temporal modeling, contrastive learning, and class-aware training, *IdentiFi* achieves accurate and robust human identification in multi-occupant indoor scenarios.

### 3.2. Spatial Discretization

**Figure 2:**
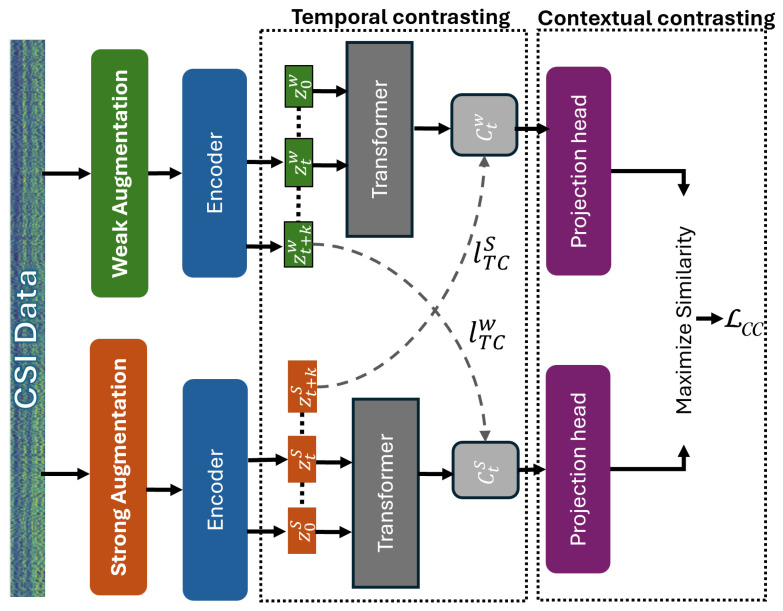
The overall architecture of the proposed contrastive learning framework. The temporal contrasting module captures robust temporal dependencies by predicting future representations from past latent features across different augmented views. The contextual contrasting module enhances feature discrimination by ensuring that representations of the same instance remain closely aligned while pushing apart representations of different instances within the mini-batch.

To facilitate both coarse localization and robust identity representation learning from Channel State Information (CSI), we adopt a spatial discretization strategy that partitions the monitored area into a structured grid of uniform cells (inspired by [54]). In our implementation, the indoor environment is virtually divided into cells measuring 1m×1m, striking a balance between spatial granularity and computational efficiency. This discretized layout enables the system to associate observed CSI patterns with approximate spatial regions, which helps reduce multipath-induced aliasing and enables better separation of overlapping signal reflections during the early stages of training. It is important to note, however, that spatial discretization in our system is not intended to serve as a high-resolution localization mechanism or to directly drive the final identity classification. Rather, it plays two auxiliary roles within the training pipeline. First, it provides localized priors to bootstrap the semi-supervised learning process through pseudo-labeling. By assigning initial weak identity labels based on users’ spatial positions, the system creates preliminary identity clusters that are then refined through contrastive learning. Second, discretization reduces the degree of signal mixing between users in widely separated regions, particularly during the early feature learning phase, where physically distant CSI patterns are less correlated due to spatial separation. We acknowledge that spatial discretization has inherent limitations—specifically, the potential for multiple individuals to occupy the same cell or for individuals to be located at the boundary of two adjacent cells. To address these cases, our system is explicitly designed to avoid relying on spatial labels during inference. The proposed contrastive learning framework shown in Figure 2 operates entirely in the latent feature space, where identity representations are learned based on personalized signal dynamics rather than spatial position. The temporal contrasting module captures dynamic features such as fine-grained motion patterns and transient signal fluctuations, which tend to be unique across individuals, even in the same spatial region. Meanwhile, the contextual contrasting module ensures that identity representations remain well separated across users while remaining consistent across augmentations. Together, these modules allow the system to form identity-specific embeddings that are resilient to spatial ambiguity, overlap, and crowding. CSI inherently contains rich information influenced by the user’s body morphology, posture, and movement, which modulates the multipath signal response in a user-specific manner. By leveraging this property, the proposed system achieves robust identity recognition without requiring high-fidelity spatial resolution. Thus, spatial discretization serves as a weak supervisory signal during training, enhancing initial signal organization, while the final identity recognition is performed entirely in a spatially invariant, contrastive latent space.

### 3.3. Data Preprocessing

The preprocessing module is a critical step in refining raw CSI data, transforming them into a structured format suitable for human identification. This process involves noise reduction, normalization, phase correction, and segmentation, ensuring that CSI signals accurately represent identity-specific features while minimizing distortions that could hinder classification. By applying a series of signal enhancement techniques, we extract clean CSI data, laying the foundation for robust feature learning. Raw CSI data are inherently susceptible to noise, which can obscure the subtle variations necessary for distinguishing individuals. To mitigate this, we employ noise reduction techniques that remove transient distortions while preserving key patterns in the signal. A moving median filter is applied to smooth out abrupt fluctuations in amplitude and phase, enhancing the stability of the signals. Additionally, phase unwrapping and offset correction are implemented to eliminate discontinuities in phase measurements, ensuring that phase variations remain coherent across different subcarriers and antennas. Once denoised, the CSI signals—originally in complex form—are decomposed into their amplitude and phase components. This separation enables targeted feature extraction, where amplitude variations capture identity-specific movement signatures, while phase variations reflect subtle shifts in signal propagation influenced by an individual’s unique physical presence. These components are then normalized using Min–Max scaling, which standardizes the data within a 0–1 range. This normalization enhances key signal structures relevant for identification, accelerates model convergence, and prevents numerical instability during training. To ensure that identity recognition remains temporally consistent, the preprocessed CSI data are segmented into fixed-length sequences that represent distinct temporal snapshots. This segmentation is designed to capture identity-linked movement dynamics, where each segment serves as an independent instance for training the AI model. By structuring data in this manner, we preserve spatial-temporal dependencies in the signal, allowing the model to learn identity-specific motion patterns across time. To further enhance the generalization capabilities of the model and improve its robustness against environmental variations, data augmentation techniques are introduced as described in the following section. This augmentation simulates realistic fluctuations in CSI signals that may arise due to environmental changes or hardware imperfections, ensuring that the system is trained to recognize identity-linked features rather than overfitting to clean training data. By incorporating controlled noise, the model learns to differentiate between intrinsic identity-related patterns and extraneous signal distortions, improving its real-world applicability.

### 3.4. Data Augmentation

Data augmentation plays a crucial role in enhancing the robustness of deep learning models trained on CSI data. Unlike conventional augmentation techniques used for images or audio, CSI data consist of both amplitude and phase components, each carrying essential spatial and temporal information about the wireless environment. Therefore, effective augmentation must consider both components to ensure realistic perturbations without corrupting the inherent physical properties of the signal.

To address this, we employ a set of augmentation techniques classified into weak and strong augmentations. Weak augmentations introduce subtle variations to the signal while preserving its structural integrity, whereas strong augmentations impose significant distortions, challenging the model to extract invariant features. Additionally, we propose advanced augmentation strategies that leverage the interplay between amplitude and phase to enhance model generalization across diverse scenarios.

#### 3.4.1. Weak Augmentations

Weak augmentations apply minimal modifications to the CSI data, allowing the model to learn robustness against minor variations.

- **Scaling** is applied independently to both amplitude and phase components to simulate variations in signal strength and environmental dynamics. Given an original CSI sample S represented as
  $$S\left[t\right]=A\left[t\right]e^{j\theta [t]},$$
  where A[t] and θ[t] denote the amplitude and phase at time *t*, respectively, we define the scaled signal as
  $$S_{\mathrm{scaled}}\left[t\right]=\alpha A\left[t\right]e^{j(\theta [t]+\beta )},$$
  where $\alpha ∼U(\alpha_{\min},\alpha_{\max})$ is a randomly sampled amplitude scaling factor, and $\beta ∼U(-\beta_{\max},\beta_{\max})$ is a small phase shift. This augmentation simulates real-world fluctuations in signal power due to environmental changes such as furniture movement.
  To further improve the model’s performance, we apply a joint augmentation strategy where a single perturbation is simultaneously applied to both amplitude and phase, preserving the signal’s structure while increasing its variability. For instance, the following scaling factor γ is introduced:
  $$S_{\mathrm{fused}}\left[t\right]=\left(\gamma A\left[t\right]\right)e^{j(\theta [t]+\gamma \beta )},$$
  where γ affects both amplitude and phase proportionally, simulating realistic signal fluctuations.
- **Jittering** introduces random noise to the CSI signal to account for small-scale variations caused by sensor imperfections. We inject independent Gaussian noise into both the amplitude and phase components:
  $$S_{\mathrm{jittered}}\left[t\right]=\left(A\left[t\right]+\eta_{A}\right)e^{j(\theta \left[t\right]+\eta_{\theta })},$$
  where $\eta_{A}∼N\left(0,\sigma_{A}^{2}\right)$ and $\eta_{\theta }∼N\left(0,\sigma_{\theta }^{2}\right)$ represent Gaussian noise for amplitude and phase, respectively. This augmentation ensures the model remains invariant to minor sensor noise and channel estimation errors.
- **Time shifting** simulates synchronization errors and delays introduced by hardware imperfections. A temporal shift Δt is randomly sampled from a uniform distribution:
  $$S_{\mathrm{shifted}}\left[t\right]=A\left[t+\Delta t\right]e^{j\theta [t+\Delta t]},$$
  where $\Delta t∼U(-T_{\max},T_{\max})$. This augmentation accounts for dynamic environmental factors that introduce phase delays, improving the model’s ability to generalize across different motion patterns.

#### 3.4.2. Strong Augmentations

Strong augmentations apply more significant perturbations, forcing the model to learn features invariant to drastic signal transformations.

- **Permutation** disrupts the temporal structure while preserving local signal dependencies. We partition the CSI sequence into *K* segments $\left\{S_{1},S_{2},\ldots ,S_{K}\right\}$ and randomly shuffle their order using a permutation function π:
  $$S_{\mathrm{permuted}}=\left\{S_{\pi (1)},S_{\pi (2)},\ldots ,S_{\pi (K)}\right\}.$$
  This augmentation encourages the model to learn local spatial–temporal features rather than relying on absolute time dependencies.
- **Time warping** introduces non-linear temporal distortions, emulating variations in movement speed and acceleration. A warping function f(t) is applied to modify the temporal axis:
  $$S_{\mathrm{warped}}\left[t\right]=A\left[f\left(t\right)\right]e^{j\theta [f(t)]},$$
  where f(t)=t+Δt(t), and $\Delta t\left(t\right)∼\mathrm{GP}(0,k\left(t,t^{\prime }\right))$ follows a Gaussian process with kernel $k(t,t^{\prime })$ ensuring smooth temporal deformations. This augmentation makes the model robust to dynamic environmental changes and variable human motion patterns.
- **Frequency masking** removes specific frequency bands, forcing the model to rely on unmasked portions of the spectrum. The masked signal is defined as
  $$S_{\mathrm{masked}}\left[f\right]=\left\{\begin{array}{cc} 0, & f\in [f_{\min},f_{\max}]\\ S[f], & \mathrm{otherwise}. \end{array}\right.$$
  This technique enhances generalization by encouraging feature extraction across multiple frequency bands.
- **MixUP** combines two different CSI samples to create augmented instances:
  $$S_{\mathrm{mixup}}=\lambda S_{i}+\left(1-\lambda \right)S_{j},$$
  where λ∼U(0,1). This method encourages the model to learn smoother decision boundaries and improves generalization across different environments.
- **Time–Frequency Transformation** applies time–frequency domain transformations, such as Short-Time Fourier Transform (STFT), and allows for spectral modifications:
  $$S_{\mathrm{stft}}=F_{\mathrm{STFT}}\left(S\right),$$
  where $F_{\mathrm{STFT}}$ represents the STFT operation. Frequency warping and stretching techniques can then be applied to augment the CSI features in the spectral domain.

#### 3.4.3. Contrastive Learning with Augmented CSI Data

The augmented CSI views form the foundation of contrastive learning. Given an original signal S, the weak and strong augmentation pipelines, $A_{w}$ and $A_{s}$, generate distinct augmented views:

$$S_{w}=A_{w}\left(S\right),\;S_{s}=A_{s}\left(S\right).$$

These views serve as inputs to a contrastive learning framework, enabling the model to learn invariant and discriminative features.

The rationale for adopting this framework lies in the complementary roles of temporal and contextual contrast. The temporal contrasting module is designed to capture identity-specific motion patterns and micro-movements encoded in the temporal structure of CSI data. These features are preserved across time windows and help form consistent representations for the same individual. On the other hand, the contextual contrasting module promotes inter-user separability by pushing representations of different users apart while pulling augmented views of the same user together. This dual mechanism enables the system to form a robust, semantically rich identity space that generalizes well to dynamic, multi-occupant environments. Together, these modules address both intra-user consistency and inter-user distinctiveness, which are essential for accurate identity recognition in real-world CSI-based sensing. By integrating these augmentation techniques, the proposed framework ensures robust CSI-based learning models capable of generalizing across varying environmental conditions and deployment scenarios.

### 3.5. The Temporal Contrasting

The temporal contrasting module employs contrastive loss to extract temporal features from CSI data in the latent space using an autoregressive model. CSI data, due to their temporal nature, capture subtle variations caused by human motion and environmental dynamics. These variations are effectively represented through latent embeddings, which preserve meaningful temporal patterns. Let $z_{t}$ denote the latent representation of the CSI signal $x_{t}$ at timestep *t*, and let $z_{\leq t}$ represent the sequence of latent representations up to time *t*.

The autoregressive model $f_{\mathrm{ar}}$ summarizes all $z_{\leq t}$ into a context vector $c_{t}$, where $c_{t}\in R^{h}$, and *h* is the hidden dimension of $f_{\mathrm{ar}}$. This context vector $c_{t}$ encapsulates the temporal dependencies of the input up to timestep *t* and is subsequently used to predict the latent representations for future timesteps $z_{t+k}$, where 1≤k≤K. To preserve mutual information between the input CSI signals and the context vector, we employ a log-bilinear model defined as

$$f_{k}\left(x_{t+k},c_{t}\right)=\exp\left({\left(W_{k}\left(c_{t}\right)\right)}^{⊤}z_{t+k}\right),$$

where $W_{k}$ is a linear projection function mapping the context vector $c_{t}$ to the same dimension as $z_{t+k}$, i.e., $W_{k}:R^{h}\to R^{d}$.

In this approach, the strong augmentation generates context vectors $c_{t}^{s}$, while the weak augmentation generates context vectors $c_{t}^{w}$. A tough cross-view prediction task is then introduced, where $c_{t}^{s}$ is used to predict the future timesteps of the weakly augmented signal $z_{t+k}^{w}$, and vice versa. The objective of the contrastive loss is to maximize the similarity between the predicted representation and the true representation of the same sample while minimizing similarity with other samples $N_{t,k}$ within the mini-batch.

The contrastive losses for the strong-to-weak prediction ($L_{TC}^{s}$) and the weak-to-strong prediction ($L_{TC}^{w}$) are formulated as

$$L_{TC}^{s}=-\frac{1}{K}\sum_{k=1}^{K}\log\frac{\exp\left({\left(W_{k}\left(c_{t}^{s}\right)\right)}^{⊤}z_{t+k}^{w}/\tau \right)}{\sum_{n\in N_{t,k}}\exp\left({\left(W_{k}\left(c_{t}^{s}\right)\right)}^{⊤}z_{n}^{w}/\tau \right)},$$

$$L_{TC}^{w}=-\frac{1}{K}\sum_{k=1}^{K}\log\frac{\exp\left({\left(W_{k}\left(c_{t}^{w}\right)\right)}^{⊤}z_{t+k}^{s}/\tau \right)}{\sum_{n\in N_{t,k}}\exp\left({\left(W_{k}\left(c_{t}^{w}\right)\right)}^{⊤}z_{n}^{s}/\tau \right)},$$

where τ is a temperature parameter, and $N_{t,k}$ denotes the set of negative samples within the batch.

In our framework, we adopt a Transformer architecture to serve as the autoregressive model due to its proven ability to efficiently capture long-range temporal dependencies in sequential data. Unlike recurrent architectures, the Transformer process sequences in parallel and is highly effective in modeling context over extended time horizons, making it well suited for CSI-based human identification tasks.

The Transformer encoder consists of *L* stacked layers, each comprising a multi-head self-attention (MHA) module followed by a feedforward multilayer perceptron (MLP) block. The MLP block includes two fully connected layers with a ReLU activation function and dropout in between. To ensure training stability and improved convergence, we employ pre-normalization residual connections throughout the architecture.

To aggregate the global temporal context, we prepend a learnable context token $c\in R^{h}$ to the sequence of input CSI features. This token acts as a global summary vector and is updated layer-by-layer alongside the temporal features. Let $z_{\leq t}\in R^{d\times T}$ denote the CSI feature sequence up to time *t*, where *d* is the input feature dimension, and *T* is the sequence length. The sequence is first projected into the hidden dimension *h* using a linear transformation $W_{\mathrm{Tran}}:R^{d}\to R^{h}$:

(1) $$\tilde{z}=W_{\mathrm{Tran}}\left(z_{\leq t}\right),\;\tilde{z}\in R^{h\times T}.$$

We then form the Transformer input by concatenating the context token to the projected features:

(2) $$\psi_{0}=\left[c;\tilde{z}\right],\;\psi_{0}\in R^{(T+1)\times h},$$

where the subscript 0 indicates the initial input to the first Transformer layer. The full filtering out self-comparisons through the following iterative computation for l=1,…,L:

(3) $$\begin{array}{cc} {\tilde{\psi }}_{l} & =\mathrm{MHA}\left(\mathrm{Norm}\left(\psi_{l-1}\right)\right)+\psi_{l-1}, \end{array}$$

(4) $$\begin{array}{cc} \psi_{l} & =\mathrm{MLP}\left(\mathrm{Norm}\left({\tilde{\psi }}_{l}\right)\right)+{\tilde{\psi }}_{l}. \end{array}$$

After processing through all layers, we extract the final representation of the context token, $c_{t}=\psi_{L}^{\left(0\right)}$, where the superscript (0) refers to the first token in the output sequence. This context vector summarizes the temporal dynamics of the input CSI sequence and is used as input to the contextual contrasting module described in Section 3.6.

This architecture enables the model to effectively encode user-specific movement patterns and spatial dynamics from raw CSI signals, thereby enhancing its capability for accurate and robust human identification across various settings.

### 3.6. Contextual Contrasting for CSI Data

To enhance the discriminative power of the learned CSI representations, we introduce a contextual contrasting module that refines identity-specific features. This module aims to learn embeddings that maximize separability across different individuals while maintaining similarity for augmented versions of the same user. To achieve this, we apply a non-linear transformation to the context vectors using a non-linear projection head, as proposed in prior work. The projection head maps the contexts into a latent space optimized for contextual contrasting.

Given a batch of *N* CSI input samples, we generate two context representations for each sample, derived from its two augmented views, leading to 2N contexts in total. For a given context $c_{t}^{i}$ corresponding to the *i*-th sample, we define its positive counterpart $c_{t}^{i+}$ as the context obtained from the other augmented view of the same sample. Hence, $\left(c_{t}^{i},c_{t}^{i+}\right)$ forms a positive pair.

Meanwhile, the remaining (2N−2) contexts, originating from different samples within the batch, are treated as negative samples. Consequently, each context $c_{t}^{i}$ forms (2N−2) negative pairs with other samples in the batch. The goal of the contextual contrasting module is to maximize the similarity between a given context and its positive counterpart while minimizing similarity with negative samples. This ensures that the final learned CSI-based representations remain robust and discriminative for human identification.

The contextual contrasting loss $L_{CC}$ is formulated as follows. Given a context $c_{t}^{i}$, its similarity with its positive counterpart $c_{t}^{i+}$ is normalized by the sum of similarities with all other (2N−1) contexts, including both the positive pair and (2N−2) negative samples:

$$\ell \left(i,i^{+}\right)=-\log\frac{\exp\left(\mathrm{sim}(c_{t}^{i},c_{t}^{i+})/\tau \right)}{\sum_{m=1}^{2N}1_{\left[m\neq i\right]}\exp\left(\mathrm{sim}(c_{t}^{i},c_{t}^{m})/\tau \right)},$$

where $\mathrm{sim}\left(u,v\right)=u^{⊤}v/∥u∥∥v∥$ represents the cosine similarity between $\ell_{2}$-normalized vectors, ensuring scale invariance in the feature space. The indicator function $1_{\left[m\neq i\right]}$ gives an evaluation of 1 if m≠i, filtering out self-comparisons. The temperature parameter τ controls the sharpness of the similarity distribution.

The overall contextual contrastive loss is computed across the batch as

$$L_{CC}=\frac{1}{2N}\sum_{k=1}^{2N}\left[\ell (2k-1,2k)+\ell (2k,2k-1)\right].$$

The final self-supervised loss integrates the two temporal contrasting losses and the contextual contrasting loss:

$$L_{\mathrm{unsup}}=\lambda_{1}\cdot \left(L_{TC}^{s}+L_{TC}^{w}\right)+\lambda_{2}\cdot L_{CC},$$

where $\lambda_{1}$ and $\lambda_{2}$ are scalar hyperparameters controlling the relative weights of the losses.

This formulation ensures that the CSI embeddings capture identity-specific spatial and temporal patterns while effectively differentiating individuals within a multi-occupant indoor environment.

### 3.7. Class-Aware Human Identification

In traditional CSI-based identification systems, classification is generally formulated as a single-label task—either recognizing a single user or detecting their presence in a fixed spatial region. However, in real-world deployments such as smart homes, healthcare facilities, or office environments, multiple users may simultaneously occupy distinct spatial regions. To address this practical challenge, we propose a novel Class-Aware Human Identification framework that formulates human sensing as a *multi-label, multi-class* problem.

In our design, the environment is discretized into a set of spatial cells C, each representing a distinct subregion. The model outputs a binary occupancy prediction ${\hat{o}}^{\left(c\right)}\in \left\{0,1\right\}$ for each cell c∈C, indicating whether the cell is occupied. For each cell predicted to be occupied, the system simultaneously performs multi-class classification over the identity space {1,…,K}, where *K* is the total number of users. This two-level hierarchy—first inferring cell occupancy, then identifying the occupant—enables fine-grained spatial reasoning and individual recognition in multi-occupant environments.

**Figure 3:**
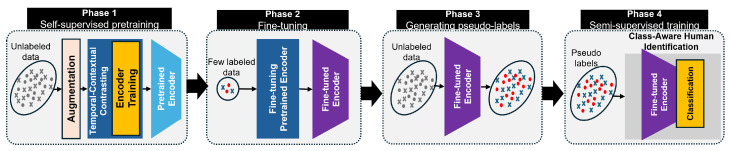
**Class-aware multi-occupant human identification framework.** The system is composed of four phases: Phase 1—self-supervised pretraining using temporal and contextual contrasting; Phase 2—supervised fine-tuning with limited labeled data; Phase 3—pseudo-label generation on unlabeled data; and Phase 4—semi-supervised training for multi-label cell occupancy detection and per-cell identity classification.

To train this model in a semi-supervised manner, we design a four-phase learning pipeline, as illustrated in Figure 3, progressively incorporating self-supervision, limited supervision, and pseudo-labeling.

**Phase 1: Self-Supervised Pretraining**. The encoder $f_{\theta }$ is first pretrained on unlabeled CSI sequences using the *temporal and contextual contrasting* (TCC) module. Each CSI sequence is augmented twice using strong and weak transformations to generate paired views, ${\hat{x}}_{2k-1}$ and ${\hat{x}}_{2k}$. These are fed into the encoder and temporal modules to perform cross-view prediction and contextual alignment. This step encourages the model to learn temporally coherent and invariant feature representations from raw CSI, without any label information.

**Phase 2: Supervised Fine-Tuning**. A small labeled dataset $D_{l}=\left\{\left(x_{k},o_{k},y_{k}\right)\right\}$ is used to fine-tune the pretrained encoder. Here, $o_{k}\in {\left\{0,1\right\}}^{\left|C\right|}$ indicates cell occupancy, and $y_{k}\in {\left\{1,\ldots ,K\right\}}^{\left|C\right|}$ specifies identity labels for each occupied cell. This phase enables the model to align its previously learned representations with task-specific signals for multi-label, multi-class discrimination.

**Phase 3: Pseudo-Label Generation**. The fine-tuned encoder is then used to generate pseudo-labels $\left(\hat{o},\hat{y}\right)$ on the unlabeled dataset $D_{u}$. For each CSI sequence, the model infers both cell-wise occupancy and user identities, thereby synthesizing weakly supervised labels that expand the training corpus.

**Phase 4: Semi-Supervised Multi-Task Learning**. Finally, the entire model is trained on both labeled and pseudo-labeled data using a multi-objective loss function. This includes (i) temporal contrastive losses, (ii) supervised contextual contrastive loss to improve cluster compactness, and (iii) cell-aware classification losses for occupancy and identity inference.

**Supervised Contextual Contrastive Learning**: To leverage label information from both manually labeled and pseudo-labeled data, we adapt contrastive learning to incorporate identity supervision. Let I={1,…,2N} denote the index set of all augmented samples. For each view i∈I, we define a set of positive matches:
  (5) $$P\left(i\right)=\left\{p\in I\setminus \left\{i\right\}\;|\;\exists c\in C,\;{\hat{o}}_{i}^{\left(c\right)}={\hat{o}}_{p}^{\left(c\right)}=1\wedge {\hat{y}}_{i}^{\left(c\right)}={\hat{y}}_{p}^{\left(c\right)}\right\},$$
  where ${\hat{o}}_{i}^{\left(c\right)}$ and ${\hat{y}}_{i}^{\left(c\right)}$ denote the predicted occupancy and identity label of cell *c* for sample *i*. The supervised contextual contrastive loss is computed as
  (6) $$L_{\mathrm{SCC}}=\sum_{i\in I}\frac{1}{\left|P(i)\right|}\sum_{p\in P(i)}\ell \left(i,p\right),$$
  where ℓ(i,p) is a contrastive loss function such as InfoNCE. This loss encourages embeddings of CSI samples that share identity and location context to be closer in the representation space.

**Cell Occupancy and Identity Classification Losses**: To train the final classification heads, we define two task-specific losses. The multi-label binary cross-entropy loss for cell occupancy is given by:
  (7) $$L_{\mathrm{occ}}=-\sum_{c=1}^{\left|C\right|}\left[o^{\left(c\right)}\log{\hat{o}}^{\left(c\right)}+\left(1-o^{\left(c\right)}\right)\log\left(1-{\hat{o}}^{\left(c\right)}\right)\right],$$
  where ${\hat{o}}^{\left(c\right)}\in \left[0,1\right]$ is the predicted probability of occupancy for cell *c*, and $o^{\left(c\right)}\in \left\{0,1\right\}$ is the ground truth.

For identity recognition in active cells, we applied categorical cross-entropy loss:

(8) $$L_{\mathrm{id}}=\sum_{c=1}^{\left|C\right|}o^{\left(c\right)}\cdot \mathrm{CE}\left(y^{\left(c\right)},{\hat{y}}^{\left(c\right)}\right),$$

where ${\hat{y}}^{\left(c\right)}$ is the predicted user distribution for cell *c*, and $y^{\left(c\right)}$ is the true user label. The multiplication by $o^{\left(c\right)}$ ensures that identity classification is only penalized for occupied cells.

**Total Training Objective**: The final objective function combines all components:
  (9) $$L_{\mathrm{total}}=\lambda_{1}\left(L_{\mathrm{TC}}^{s}+L_{\mathrm{TC}}^{w}\right)+\lambda_{2}L_{\mathrm{SCC}}+\lambda_{3}\left(L_{\mathrm{occ}}+L_{\mathrm{id}}\right),$$
  where $\lambda_{1},\lambda_{2},\lambda_{3}\in R^{+}$ are tunable hyperparameters that balance the contributions of contrastive pretraining, cluster consistency, and the final classification objectives.

To the best of our knowledge, this is the first work that formulates CSI-based human identification as a hierarchical multi-label, multi-class problem—enabling the simultaneous detection and recognition of multiple users across spatial cells. Our integration of contrastive pretraining, label-efficient fine-tuning, and class-aware representation learning bridges the gap between single-user assumptions and real-world, multi-occupant deployments.

## 4. Evaluation

### 4.1. Experimental Setup and Data Collection

**Figure 4:**
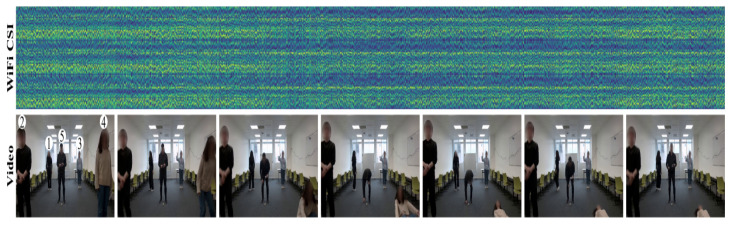
An example showing the participants practicing different activities. User 1: rotation. User 2: jumping. User 3: waving. User 4: lying down. User 5: picking up [55].

To assess the effectiveness of our system, we conducted experiments using a publicly available dataset [55] collected in a real-world indoor setting as shown in Figure 4. The experiments were designed to capture WiFi Channel State Information (CSI) associated with human identification in multi-occupant scenarios. Participants performed activities either simultaneously or individually, allowing us to evaluate the system’s ability to distinguish identities based on their unique movement patterns.

The dataset comprises 11,286 CSI samples, each synchronized with video recordings, collectively spanning approximately 9.4 h of data. These recordings were used to establish ground truth for human identity verification.

**Hardware and CSI Configuration**: To collect CSI data, two standard computing devices (HP EliteDesk 800 G2 TWR, manufactured by Hewlett-Packard Inc., Palo Alto, CA, USA) were deployed as the transmitter and receiver. Both were equipped with an (manufactured by Intel Corp., Santa Clara, CA, USA) and operated using the Linux 802.11n CSI tool. The wireless communication was configured to use two frequency bands, channel 12 in the 2.4 GHz band and channel 64 in the 5 GHz band, enabling dual-band CSI collection.

The CSI acquisition process was conducted in three sequential steps. First, the receiver was configured to capture CSI from all received packets while monitoring a specific WiFi channel. Next, the transmitter continuously sent packets over the channel while participants moved naturally or performed specific activities. This ensured that each participant’s unique movement signature was reflected in the collected CSI data. Once data collection was complete, the receiver stopped logging, marking the end of each recording session.

In accordance with best practices in WiFi sensing [56,57], each recording session lasted for 3 s, during which the transmitter sent 3000 packets at a rate of 1000 packets per second. Consequently, each CSI sample contained 3000 time steps. The data were recorded using three antennas, each receiving signals across 30 subcarriers, resulting in a CSI matrix of dimensions 3×3×30 per time step. Thus, each CSI sample consisted of a high-dimensional tensor of shape 3000×3×3×30, preserving the spatial and temporal characteristics necessary for human identification.

**Figure 5:**
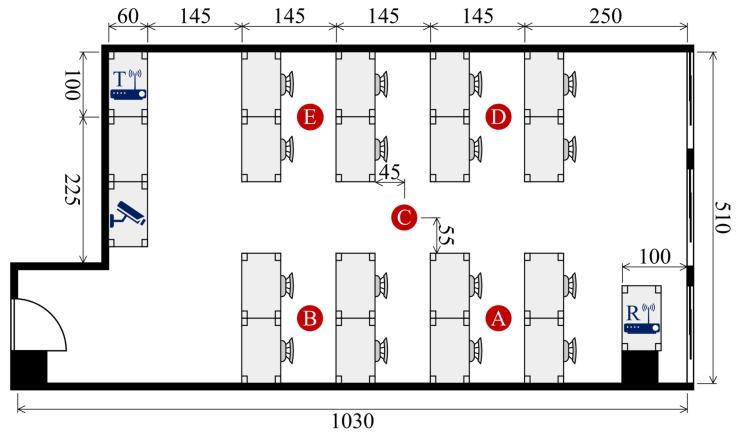
Classroom environment [55].

**Figure 6:**
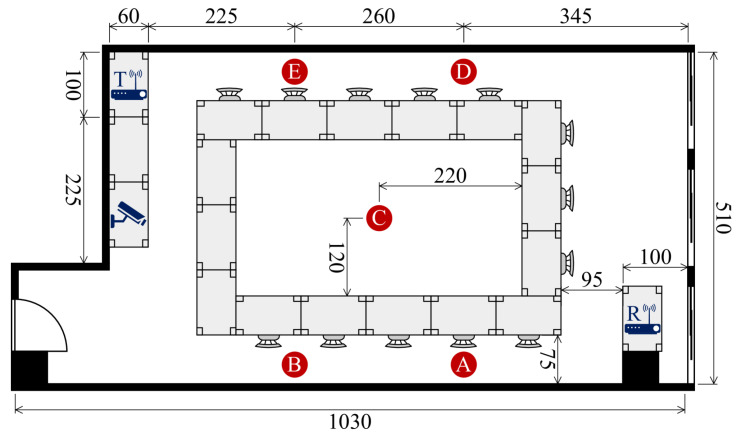
Meeting environment [55].

**Figure 7:**
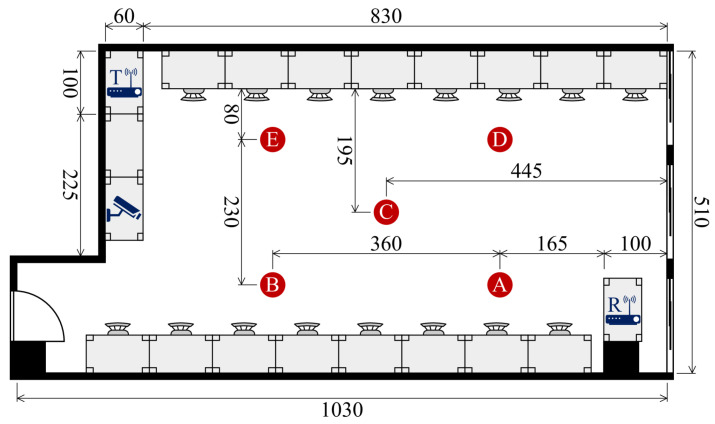
Empty environment [55].

**Participants and Experimental Environments**: Data were collected across three distinct indoor environments: a classroom (Figure 5), a meeting room (Figure 6), and an empty room (Figure 7). Each environment had five predefined positions where participants were stationed or moved during data collection. A total of six individuals (three males and three females) participated in the experiment, contributing to a diverse dataset for identity recognition.

**Data Collection Protocol**: To ensure consistency and realism, participants performed natural human activities such as standing, sitting, walking, and other common indoor movements. These activities influenced CSI in a way that allowed us to model unique movement signatures for each person. Each recording session was accompanied by synchronized video recordings, providing precise ground-truth labels for identity verification.

### 4.2. Semi-Supervised Training for CSI-Based Human Identification

**Figure 8:**
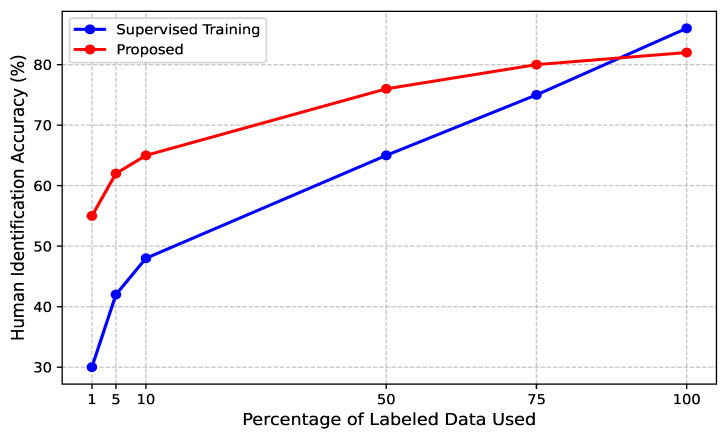
Comparison between supervised training and the proposed semi-supervised learning approach of *IdentiFi* for different few-labeled data scenarios.

To assess the effectiveness of the proposed contrastive learning framework for CSI-based human identification under semi-supervised conditions, we trained the model using different proportions of labeled data: 1%, 5%, 10%, 50%, 75%, and 100% of the total training set. Figure 8 illustrates the comparative performance between the proposed contrastive learning approach and conventional supervised training across these varying label availability settings. Specifically, the fine-tuning process (represented by the red curve in the figure) involved refining the pre-trained encoder using a limited set of labeled CSI samples. Our results demonstrate that traditional supervised learning exhibits significant performance degradation when trained with limited labeled data, whereas the proposed semi-supervised contrastive fine-tuning strategy consistently achieves superior accuracy, even with minimal labeled samples. Notably, with only 1% labeled CSI data, the fine-tuned model attains approximately 55% accuracy, whereas the fully supervised model lags behind at 30% accuracy, showcasing the model’s ability to generalize with minimal supervision. As the proportion of labeled data increases, the contrastive fine-tuning approach continues to outperform supervised training. For instance, with 10% labeled data, the proposed model achieves an accuracy of 65%, matching the performance of a supervised model trained on 50% labeled data. When trained with 50% labeled data, the fine-tuned model reaches 76% accuracy, significantly surpassing its supervised counterpart at the same label ratio. Most notably, the contrastive learning framework trained with only 75% labeled CSI data achieves accuracy levels comparable to fully supervised training with 100% labeled data. This highlights the effectiveness of leveraging self-supervised pretraining on CSI signals, enabling the model to learn identity-specific movement signatures even when ground-truth labels are scarce. These findings affirm the suitability of the proposed semi-supervised approach for practical human identification applications, particularly in scenarios where manually labeling CSI data is infeasible.

### 4.3. Effectiveness of Temporal and Contextual Contrasting

**Figure 9:**
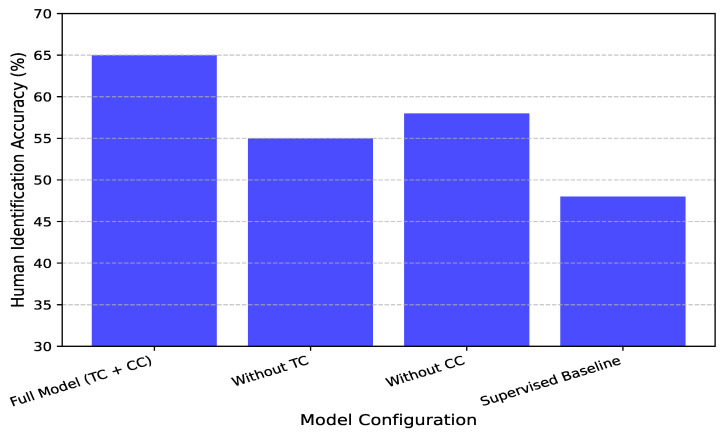
Performance comparison of different human identification systems in an empty room environment.

To assess the impact of the proposed temporal contrasting (TC) and contextual contrasting (CC) modules on CSI-based human identification, we conducted controlled experiments to analyze their contributions to identity-specific representation learning. We compared models trained with both modules enabled against ablated versions where one or both components were removed. Figure 9 illustrates the degradation in identification performance when temporal contrasting is omitted, particularly in low-label scenarios. With only 10% labeled data, the full model achieves 65% accuracy, whereas the version trained without TC drops to 55%, underscoring the role of temporal consistency in capturing distinct movement patterns. Compared to a fully supervised model, which also achieves 55% in this setting, our contrastive learning approach demonstrates superior generalization, emphasizing the significance of enforcing consistency in latent representations across augmented views. Similarly, the exclusion of contextual contrasting results in a substantial performance drop, especially in multi-user environments. As shown in Figure 9, with 10% labeled data, the full model attains 65% accuracy, while the absence of CC reduces accuracy to 58%, highlighting the necessity of contrastive learning to distinguish individuals effectively. The supervised baseline, again at 55%, further validates the benefits of this contrastive approach. Contextual contrasting ensures identity-specific representations remain well separated in the embedding space by enforcing inter-class separation among different individuals’ CSI features. These findings confirm that temporal contrasting captures essential temporal dependencies, while contextual contrasting enhances inter-class separability, both of which are critical for robust CSI-based human identification. The combination of these modules significantly improves performance, particularly when labeled data are scarce.

### 4.4. Effect of Data Augmentation Techniques

**Figure 10:**
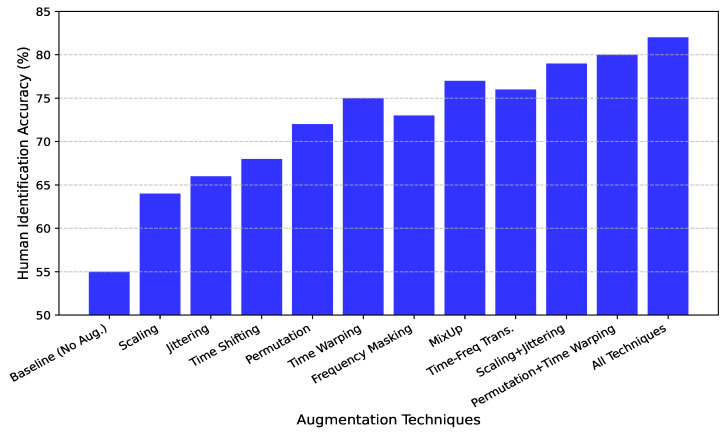
Impact of data augmentation techniques on CSI-based human identification accuracy.

Data augmentation is a critical component in contrastive learning frameworks, especially in the context of CSI-based human identification where signal characteristics are highly sensitive to environmental dynamics, device placement, and user behavior. Augmentation techniques serve as an implicit form of regularization by exposing the model to a wider variety of transformations during training, thereby forcing it to learn more invariant and generalizable representations. In this section, we provide a comprehensive analysis of several augmentation strategies—ranging from weak, structure-preserving transformations to strong, structure-altering perturbations—and their influence on model performance as shown in Figure 10.

We begin by analyzing the role of weak augmentations, which apply modest alterations to the input signal. These transformations are designed to maintain the semantic identity and spatiotemporal structure of the original signal while introducing realistic noise or minor distortions. Among these, amplitude scaling, jittering, and time shifting prove particularly effective. Scaling slightly varies the amplitude of the CSI signal, simulating changes in signal strength caused by minor body movements or hardware-level fluctuations. Jittering adds small amounts of random noise to simulate environmental interference, while time shifting emulates small temporal misalignments, such as those resulting from asynchronous sampling or latency in the system. Empirically, these augmentations lead to steady improvements in identification accuracy, reaching 64%, 66%, and 68% respectively—representing gains of 9%, 11%, and 13% over the 55% baseline. These results indicate that even subtle augmentations help the model develop robustness to signal-level variations without compromising its ability to learn person-specific features.

On the other hand, strong augmentations are designed to significantly alter the temporal or spectral structure of the CSI signals, challenging the model to extract deeper, more abstract representations that remain consistent across drastically modified views. Permutation and time warping exemplify this class. Permutation breaks the temporal order of the signal in controlled segments, while time warping non-linearly stretches or compresses temporal intervals. Despite the aggressive nature of these transformations, the model not only maintains its discriminative capacity but actually improves upon it, achieving identification accuracies of 72% and 75%, corresponding to 17% and 20% absolute gains. This suggests that forcing the model to handle temporally distorted input promotes learning representations that are less reliant on exact sequence ordering and more focused on semantic structure.

Further improvements are observed with frequency masking, which randomly suppresses the frequency components of the input CSI sequence. This technique mimics hardware-level interference or frequency band dropout, forcing the model to learn redundant representations that do not overly depend on any single spectral region. The model achieves 73% accuracy under frequency masking, with an 18% improvement over the baseline. Notably, the strongest performance among all individual techniques is observed with MixUp, a data interpolation method that blends two CSI samples and their corresponding labels to create virtual training examples. This technique regularizes the model by smoothing the decision boundaries between classes and reducing overfitting. The resulting accuracy of 77%—a 22% improvement—demonstrates the strong regularization benefit introduced by this synthetic data generation. These results show that the best performance is achieved when augmentation techniques are used in combination. The joint application of scaling and jittering, two weak augmentations that complement each other, boosts accuracy to 79%. Similarly, combining permutation and time warping—two strong, temporally focused augmentations—achieves 80% accuracy. These results confirm that augmentation strategies affecting different signal domains (e.g., amplitude vs. temporal) can work synergistically to enhance the model’s robustness. When all augmentation techniques are applied simultaneously, the model achieves its highest accuracy of 82%, a substantial 27% improvement over the baseline. This result underscores a key insight: diversity in augmentation is as crucial as augmentation strength. By exposing the model to a wide range of transformations—across time, frequency, and amplitude domains—we enable it to internalize generalizable patterns that remain discriminative across users, environmental conditions, and deployment scenarios. Taken together, these findings validate our design choice of incorporating both weak and strong augmentations into the training pipeline. These results demonstrate that even in the absence of labeled data, carefully crafted transformations allow the contrastive learning framework to learn robust identity-aware representations. The clear upward trend in accuracy with increasingly rich and diverse augmentation strategies confirms their essential role in enhancing the performance, generalization, and practical deployability of CSI-based human identification systems.

### 4.5. Performance Across Different Frequency Bands in Different Environments

**Figure 11:**
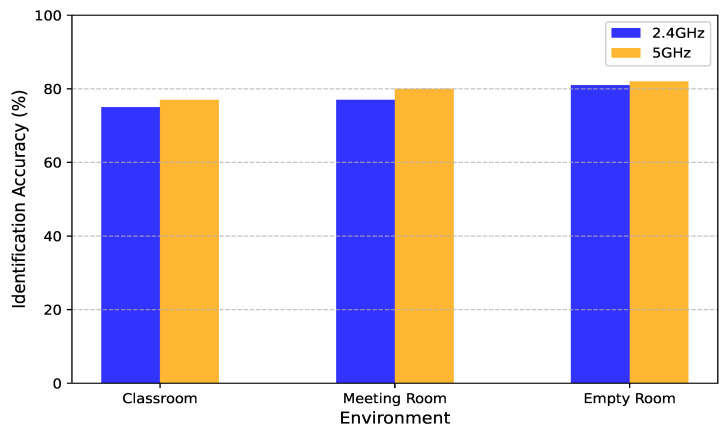
Activity recognition performance of *IdentiFi* in different frequency settings.

The operating frequency band plays a fundamental role in determining the quality and informativeness of CSI measurements. As WiFi-based CSI sensing systems become increasingly deployed in real-world spaces, understanding how different frequency bands—specifically 2.4 GHz and 5 GHz—affect identification accuracy in varying environments is crucial for robust system design and deployment. We evaluate the human identification performance of our system across three distinct indoor environments: a classroom, a meeting room, and an empty room. Each space presents a unique set of propagation characteristics, including multipath richness, signal attenuation, and interference levels. Figure 11 illustrates the identification accuracies achieved at 2.4 GHz and 5 GHz across these environments.

Our results show that the system maintains consistently high performance across both frequency bands and all environments, reflecting the robustness and generalizability of the learned representations. However, a consistent pattern emerges: the 5 GHz band outperforms 2.4 GHz in every setting, albeit with varying margins. In the classroom environment, accuracy improves from 75% at 2.4 GHz to 77% at 5 GHz. In the meeting room, the improvement is more notable, rising from 77% to 80%. The most modest gain is observed in the empty room, where accuracy increases from 81% to 82%. Although the absolute improvements may seem small, they are consistent and statistically meaningful, particularly given the already high baseline performance.

The superior performance of the 5 GHz band can be attributed to its distinct physical and spectral characteristics. First, the higher bandwidth of 5 GHz facilitates finer-grained CSI measurements by increasing subcarrier density, thus enabling more detailed tracking of motion-induced variations. This granularity is especially important in human identification, where subtle differences in body movement patterns and gaits must be captured to reliably distinguish between individuals.

Second, the 5 GHz spectrum is typically less congested than the 2.4 GHz band, which is shared by many devices, including microwaves, Bluetooth peripherals, and IoT appliances. Reduced spectral interference at 5 GHz results in cleaner, more stable signal measurements, which directly benefit the reliability of CSI features and downstream learning processes. This is particularly impactful in busy environments like classrooms or offices, where signal collisions and overlapping channels are common in the 2.4 GHz band.

Third, the shorter wavelength of 5 GHz signals enhances the sensitivity of the system to small-scale movements and spatial changes. This property allows the system to better resolve fine-grained variations in user posture, gait, and gesture, which are critical for identity discrimination. In more cluttered or occluded environments—such as meeting rooms filled with furniture and equipment—this increased spatial resolution leads to a more pronounced performance advantage over 2.4 GHz.

Interestingly, the performance gap between the two bands is smallest in the empty room setting. This is expected, as the absence of obstacles and minimal interference provide an ideal propagation environment, allowing both bands to achieve near-maximum sensing effectiveness. In contrast, the more complex propagation dynamics in furnished or crowded environments amplify the benefits of 5 GHz’s cleaner spectrum and higher spatial resolution.

Overall, these findings highlight the importance of frequency band selection in CSI-based sensing applications. While our system demonstrates strong cross-band generalization, the use of 5 GHz signals provides a measurable boost in accuracy, particularly in environments with high interference potential or complex spatial layouts. These insights have practical implications for system deployment, suggesting that leveraging dual-band sensing or favoring 5 GHz when available can enhance performance in real-world multi-user settings such as smart classrooms, conference rooms, and healthcare facilities.

### 4.6. The Effect of Changing the Cell Size

**Figure 12:**
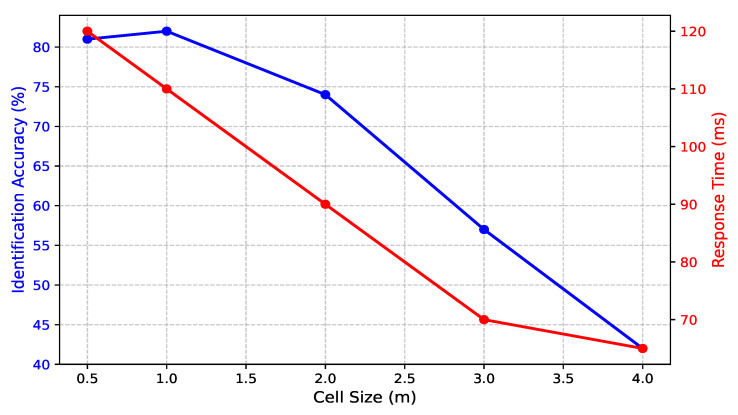
Impact of cell size on identification accuracy and response time of *IdentiFi*.

This section investigates how varying cell size affects the performance of CSI-based human identification, considering both identification accuracy and response time. As illustrated in Figure 12, the choice of cell size plays a critical role in system efficiency and precision. When using a small cell size (0.5 m), the system achieves 81% accuracy; however, this fine spatial resolution introduces challenges. Individuals may occupy multiple adjacent cells simultaneously, leading to ambiguity in identification. Furthermore, processing a larger number of cells significantly increases computational complexity, causing the response time to rise to 120 ms. Although the accuracy remains high, the increased latency makes such small cell sizes impractical for real-time applications.

As the cell size increases to 1.0 m, the system attains its best balance between accuracy and efficiency, with 82% accuracy and a reduced response time of 110 ms. At this scale, spatial granularity is sufficient to differentiate individuals while maintaining a manageable computational workload. This configuration ensures that identification remains precise without overwhelming system resources. Beyond 1.0 m, further increases in cell size lead to a progressive decline in accuracy. At 2.0 m, accuracy drops to 74%, and at 3.0 m, it further declines to 57%. This decline is due to spatial overlap, where multiple individuals share the same cell, reducing the system’s ability to distinguish unique movement patterns. While response time improves to 90 ms at 2.0 m and 70 ms at 3.0 m, the compromise in identification precision limits the practical usability of these larger cell sizes. With a cell size of 4.0 m, the system achieves the lowest accuracy of 42%, despite achieving the fastest response time of 65 ms. The significant loss in spatial resolution prevents the system from effectively distinguishing between different individuals, making this configuration unsuitable for accurate identification. These results confirm that cell size is a crucial factor in balancing system performance and efficiency. The optimal cell size of 1.0 m achieves the highest accuracy (82%) while maintaining a reasonable response time (110 ms). This configuration ensures a practical trade-off between precision and real-time processing, making it an effective choice for CSI-based human identification in real-world applications.

### 4.7. The Effect of Increasing the Number of Persons in the Environment

**Figure 13:**
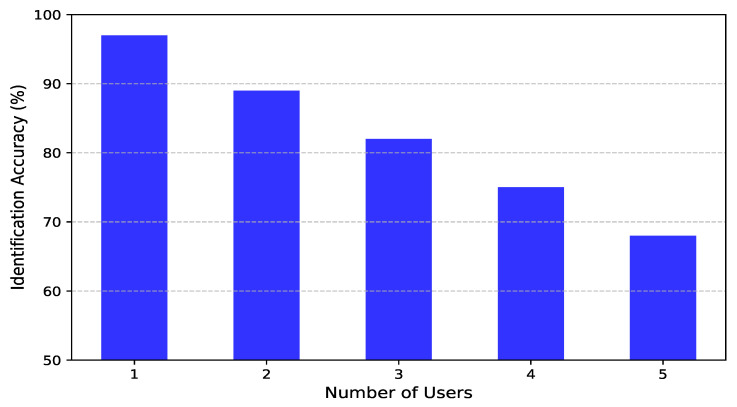
The effect of changing the number of users in the environment on *IdentiFi*’s performance.

This section examines how the presence of multiple individuals in the environment affects the system’s identification accuracy. As depicted in Figure 13, the system demonstrates high accuracy when the number of users is limited. With a single user, identification accuracy reaches 97%, and it remains high at 89% and 82% for two and three users, respectively. However, as the number of individuals increases, accuracy gradually declines, reaching 75% for four users and 68% for five users. Despite this decline, the system maintains strong performance, underscoring its resilience in multi-user settings. This robustness is primarily attributed to the contrastive learning framework and advanced data augmentation techniques incorporated into the training process. The contrastive learning approach enables the system to extract identity-specific features while minimizing the impact of signal interference from multiple users. By enforcing similarity between different augmented views of the same individual while pushing apart representations of different individuals, the model learns highly discriminative features that remain effective even in complex environments.

Additionally, the use of diverse augmentation strategies—such as time warping, frequency masking, and MixUp—enhances the model’s ability to generalize to challenging scenarios. These techniques introduce controlled variations in the CSI data, simulating real-world fluctuations in signal propagation caused by multiple occupants. This augmentation-driven robustness allows the system to adapt to dynamic environments, reducing performance degradation as the number of users increases. While the presence of more users naturally introduces greater signal complexity, the system’s ability to sustain an accuracy above 68% in such scenarios highlights its practical applicability. This confirms its suitability for real-world deployment in environments such as smart buildings, security systems, and healthcare monitoring, where accurate identification in multi-user settings is essential.

### 4.8. Comparative Analysis of Human Identification Performance

To assess the effectiveness of the proposed system, we conducted a comparative evaluation against a comprehensive set of well-established baseline models that utilize WiFi-based CSI data for human identification. These baselines encompass traditional machine learning techniques, deep neural networks, and a self-supervised learning approach. Specifically, the comparative models include Short-Time Fourier Transform-based Random Forest (ST-RF) [41], multilayer perceptron (MLP) [14], Long Short-Term Memory (LSTM) [41], a one-dimensional Convolutional Neural Network (CNN-1D) [14], a two-dimensional Convolutional Neural Network (CNN-2D) [43], Convolutional LSTM (CLSTM) [48], Attention-Based LSTM (ABLSTM) [7], a Temporal Hierarchical Attention Network (THAT) [58], and a variant of SimCLR [59] adapted to CSI signals for self-supervised learning.

The ST-RF approach extracts time–frequency domain features using Short-Time Fourier Transform and applies a Random Forest classifier. While straightforward, this method relies heavily on manual feature engineering and struggles to capture fine-grained motion-induced signal characteristics. MLP, as a fully connected feedforward neural network, treats CSI inputs as flattened vectors, failing to exploit any inherent sequential or spatial structure in the data. LSTM, a recurrent neural network architecture, improves upon this by modeling temporal dependencies, yet it lacks spatial awareness and often underperforms in noisy or complex environments. CNN-1D and CNN-2D architectures are employed to extract local features along temporal and spatial dimensions, respectively. However, their receptive fields are limited, and they do not capture longer-term dependencies effectively. CLSTM attempts to address this by combining convolutional operations with temporal memory mechanisms, offering a more balanced spatial–temporal representation. ABLSTM introduces an attention mechanism atop LSTM to dynamically weight temporal features, but its flat attention structure is insufficient for hierarchical modeling. THAT enhances temporal modeling further through a hierarchical Transformer-based attention design, representing the most sophisticated of the prior baselines. To further strengthen the evaluation, we included SimCLR, a general-purpose contrastive learning framework, adapted here for CSI-based identification. In this adaptation, different temporal augmentations of the same CSI sequence are treated as positive pairs, while sequences from different users are treated as negatives. This model was trained without labels, leveraging contrastive loss to learn meaningful identity-preserving features from unlabeled data.

Identification accuracy was employed as the primary evaluation metric, consistent with prior research [7,41,58], to quantify system performance across different models. The dataset was partitioned into an **80% training set** and a **20% test set**, ensuring a fair and consistent comparison across all methods.

**Figure 14:**
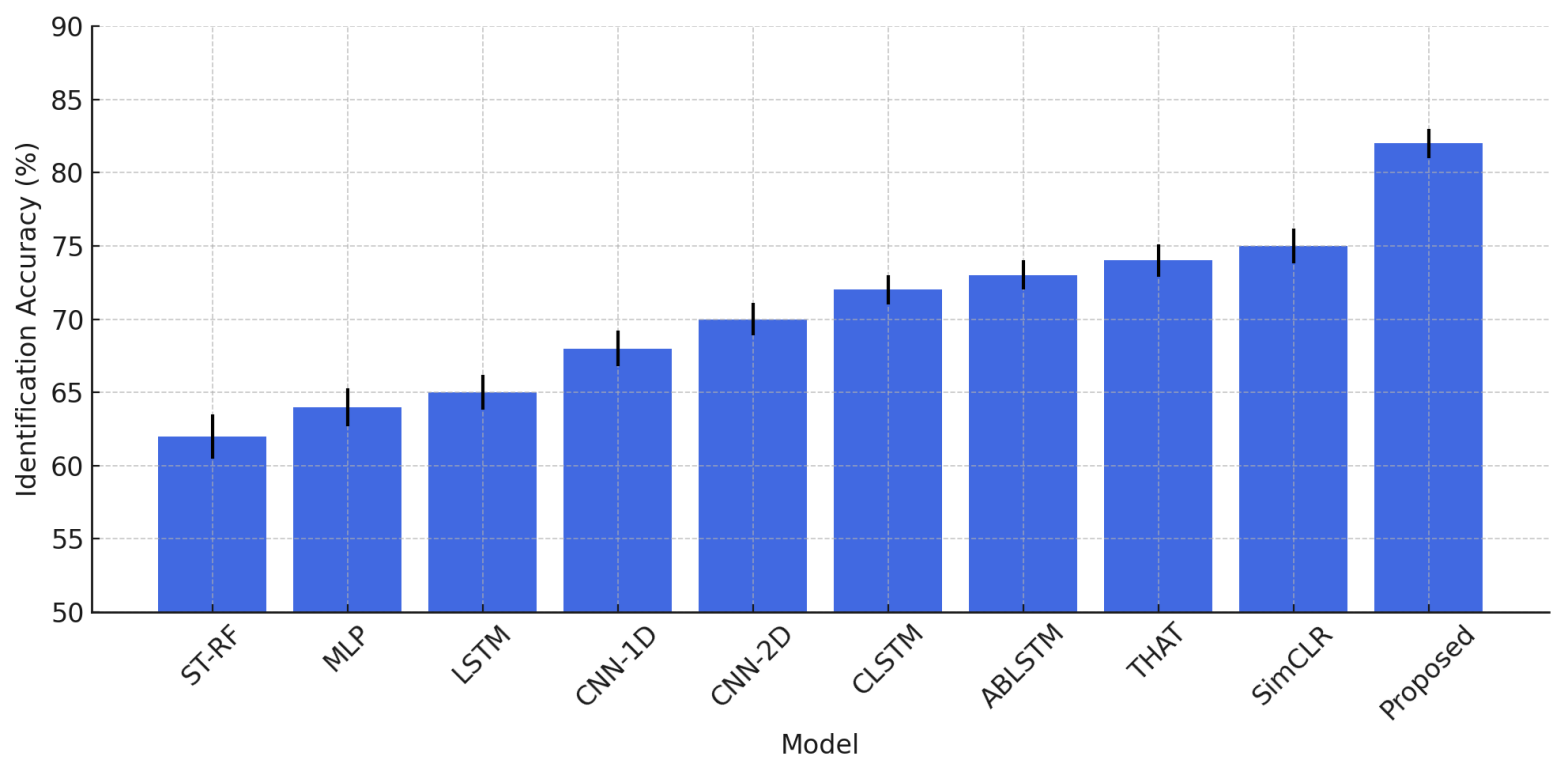
Identification accuracy comparison of proposed system with baseline models.

Figure 14 illustrates the identification accuracy of the proposed system compared to existing models. The proposed system achieves an accuracy of **82%**, significantly surpassing the baseline methods. Traditional machine learning models such as ST-RF and MLP exhibit the lowest performance, achieving **62%** and **64%**, respectively, due to their reliance on handcrafted features and inability to model temporal–spatial dynamics in CSI data. Neural network-based models, including LSTM (**65%**), CNN-1D (**68%**), and CNN-2D (**70%**), demonstrate moderate improvements by capturing sequential or local spatial patterns, but are still limited in handling complex human motion variations. CLSTM and ABLSTM provide additional gains, reaching **72%** and **73%**, respectively, by better modeling temporal dependencies and incorporating attention. THAT advances further by introducing hierarchical temporal attention, achieving **74%**, and serves as the strongest deep learning baseline. The SimCLR model attains **75%**, outperforming all supervised baselines. This result confirms the promise of contrastive learning in this domain, particularly in low-label scenarios. However, its general-purpose nature limits its ability to leverage the unique temporal and contextual structures embedded in CSI data.

In contrast, the proposed *IdentiFi* system demonstrates superior performance due to its integrated Transformer-based architecture and domain-specific learning modules. The temporal contrasting module captures evolving motion patterns unique to each individual, while the contextual contrasting module ensures identity separability in multi-user settings. Unlike conventional models, IdentiFi processes CSI data in a multi-view and contrastive fashion, enabling it to learn fine-grained identity-specific features even with limited supervision. Moreover, the system’s robustness is evident in its ability to handle overlapping signal sources, which degrade the performance of other methods. Through contextual contrasting, the proposed system effectively disentangles identity embeddings despite inter-user signal interference. The inclusion of both self-supervised and semi-supervised training strategies further enhances generalization and reduces reliance on labeled data, a significant advantage for real-world deployment.

These results confirm that IdentiFi establishes a new state of the art in CSI-based human identification. Its significant accuracy improvement, combined with its robust performance in complex, dynamic, and multi-occupant environments, underlines its practicality and scalability for deployment in smart sensing applications.

### 4.9. Comparative Analysis of Training Time and Real-Time Inference Efficiency

In addition to identification accuracy, it is essential to assess the computational efficiency of human identification models, particularly in the context of real-time deployment within smart environments. We evaluated and compared all baseline models and the proposed *IdentiFi* system in terms of both **training time** and **inference latency**, using a high-performance workstation equipped with dual Intel Xeon Silver 4210 CPUs (Intel Corp., Santa Clara, CA, USA), 320 GB RAM, and NVIDIA RTX A6000 GPU (NVIDIA Corp., Santa Clara, CA, USA) Training time was measured as the duration (in minutes) required for model convergence under consistent conditions, with an **80% training** and **20% testing** dataset split. Inference latency was recorded per CSI sample in milliseconds and reflects the system’s ability to respond in online scenarios. These metrics are particularly relevant for time-sensitive human-centered applications such as indoor security, smart home personalization, and responsive ambient systems.

**Figure 15:**
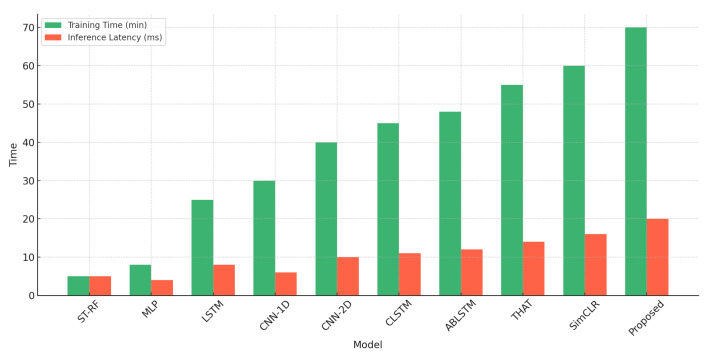
Comparisonof training time (minutes) and inference latency (milliseconds) across baseline models and the proposed system.

Figure 15 illustrates a comparison of the models’ computational performance. Traditional models like ST-RF and MLP offer minimal training time (5–8 min) and sub-5 ms inference latency due to their shallow architectures and static feature representations. However, this efficiency comes at the cost of significantly lower accuracy, as they fail to model the nuanced temporal and spatial dynamics embedded in CSI data. Recurrent and Convolutional Neural Networks—including LSTM, CNN-1D, and CNN-2D—require moderate training durations (25–40 min) and achieve low-latency inference (6–10 ms), but their limited capacity to capture cross-channel and inter-frame dependencies limits their accuracy in complex environments. CLSTM and ABLSTM extend these models by incorporating either convolutional filtering or attention mechanisms, leading to slightly longer training times (45–48 min) and inference latencies of 11–12 ms. THAT and SimCLR represent more sophisticated baselines. THAT, with its hierarchical attention structure, trains in 55 min and operates with a 14 ms latency. SimCLR, as a contrastive self-supervised framework adapted to CSI, requires 60 min to train and 16 ms for inference, benefiting from data efficiency but lacking task-specific architectural tailoring.

The proposed *IdentiFi* system introduces the highest training overhead at **70 min**, owing to its composite architecture integrating a Transformer backbone with temporal and contextual contrastive learning modules. However, this additional training investment results in the most significant accuracy gains across all models. Importantly, the system maintains a highly competitive inference latency of only **20 ms**, comfortably within the real-time threshold for smart environment responsiveness. This performance is made possible by several factors: the architectural optimization of multi-head attention, the elimination of contrastive loss evaluation during inference, and the efficient GPU-parallel execution of Transformer layers. Even with its rich modeling depth and semi-supervised learning components, *IdentiFi* operates efficiently during deployment, ensuring that high accuracy does not come at the cost of responsiveness.

These results highlight that *IdentiFi* not only achieves state-of-the-art accuracy but also maintains real-time viability, making it exceptionally well suited for practical deployment in latency-sensitive, multi-occupant, dynamic environments. Its ability to balance learning complexity with operational speed positions it as a robust and scalable solution for next-generation human identification systems.

## 5. Conclusions

In conclusion, the proposed *IdentiFi* system represents a significant advancement in the field of human identification within smart environments. By leveraging WiFi-based wireless sensing, self-supervised contrastive learning, and semi-supervised fine-tuning, *IdentiFi* addresses key challenges associated with existing vision-based and WiFi-based human identification methods. Its ability to accurately distinguish between individuals in multi-occupant environments while preserving privacy makes it particularly suited for applications in smart homes, healthcare, security, and personalized services. Through robust temporal and contextual contrasting modules, as well as innovative augmentation techniques, *IdentiFi* ensures reliable identification even under varying environmental conditions. The system’s efficient use of unlabeled data through self-supervised learning further enhances its scalability and practicality. As demonstrated through comprehensive evaluations, *IdentiFi* establishes a new standard for adaptive, privacy-preserving human identification, paving the way for more intelligent and human-centric smart environments.

## Data Availability

Data are contained within the article.
